# Development and Validation of a Novel Risk Stratification Model for Cancer-Specific Survival in Diffuse Large B-Cell Lymphoma

**DOI:** 10.3389/fonc.2020.582567

**Published:** 2021-01-14

**Authors:** Qiaofeng Zhong, Yuankai Shi

**Affiliations:** Department of Medical Oncology, National Cancer Center/National Clinical Research Center for Cancer/Cancer Hospital, Chinese Academy of Medical Sciences & Peking Union Medical College, Beijing Key Laboratory of Clinical Study on Anticancer Molecular Targeted Drugs, Beijing, China

**Keywords:** diffuse large B-cell lymphoma, cancer-specific survival, SEER, prognosis, risk stratification

## Abstract

Diffuse large B-cell lymphoma (DLBCL) is a biologically and clinically heterogenous disease. Identifying more precise and individual survival prognostic models are still needed. This study aimed to develop a predictive nomogram and a web-based survival rate calculator that can dynamically predict the long-term cancer-specific survival (CSS) of DLBCL patients. A total of 3,573 eligible patients with DLBCL from 2004 to 2015 were extracted from the Surveillance, Epidemiology and End Results (SEER) database. The entire group was randomly divided into the training (n = 2,504) and validation (n = 1,069) cohorts. We identified six independent predictors for survival including age, sex, marital status, Ann Arbor stage, B symptom, and chemotherapy, which were used to construct the nomogram and the web-based survival rate calculator. The C-index of the nomogram was 0.709 (95% CI, 0.692–0.726) in the training cohort and 0.700 (95% CI, 0.671–0.729) in the validation cohort. The AUC values of the nomogram for predicting the 1-, 5-, and 10- year CSS rates ranged from 0.704 to 0.765 in both cohorts. All calibration curves revealed optimal consistency between predicted and actual survival. A risk stratification model generated based on the nomogram showed a favorable level of predictive accuracy compared with the IPI, R-IPI, and Ann Arbor stage in both cohorts according to the AUC values (training cohort: 0.715 *vs* 0.676, 0.652, and 0.648; validation cohort: 0.695 *vs* 0.692, 0.657, and 0.624) and K-M survival curves. In conclusion, we have established and validated a novel nomogram risk stratification model and a web-based survival rate calculator that can dynamically predict the long-term CSS in DLBCL, which revealed more discriminative and predictive accuracy than the IPI, R-IPI, and Ann Arbor stage in the rituximab era.

## Introduction

Diffuse large B-cell lymphoma (DLBCL) is the most common type of aggressive lymphoma, accounting for 30–40% of all newly diagnosed non-Hodgkin lymphomas (NHL) (1). It is a heterogeneous group of lymphomas in terms of clinical presentation, tumor biology and prognosis (2). The addition of rituximab to conventional CHOP chemotherapeutic regimen (cyclophosphamide, doxorubicin, vincristine, and prednisone) has dramatically improved the treatment outcome of patients with DLBCL (3). However, approximately 30–40% of patients still experience disease relapse or refractory to therapy (4).

The International Prognostic Index (IPI) (5) and its subsequent revisions [Revised‐IPI (R‐IPI)] (6) and National Comprehensive Cancer Network IPI (NCCN-IPI) (7) remain the most useful prognostications for DLBCL. These scoring systems were based on similar factors: patient age, Eastern Cooperative Oncology Group (ECOG) performance status (PS), Ann Arbor stage, serum lactate dehydrogenase (LDH), and extranodal involvement. IPI is the first, best validated, and still most widely used tool for routine clinical application in DLBCL. However, it was developed far before the introduction of rituximab in the clinical practice, and its discriminating power has decreased in the rituximab era. R‐IPI was based on a cohort including more patients with younger age, it may be of limited value for elderly patients. NCCN‐IPI has been shown to provide better risk stratification than the IPI, nonetheless, it was still insufficiently accurate to be applied in clinical practice.

Hence, novel more precise prognostic tools are needed to assess patients’ prognosis in the rituximab era. A variety of molecular biomarkers and gene-based predictors have good discrimination ability (8–14). However, these methods are costly, technically challenging in developing countries and require further validation. Therefore, identifying new, easily accessible and more accurate prognostic markers is still needed.

By integrating various significant factors, a nomogram can predict and quantify the probability of individual patient developing a certain clinical event, such as the possibility of disease death or recurrence. In addition, it had shown a more effective predicted ability than traditional staging systems in several cancers. Therefore, the nomogram has become an important instrument for clinical decision making and risk stratification in oncology (15). Although nomogram can improve predictive accuracy, it may be difficult to use in clinical practice due to need to perform manual calculations. The web-based calculator based on nomogram allows to enhances calculation efficiency of CSS rate.

Therefore, the current study aimed to develop a predictive nomogram and a web-based survival rate calculator that can dynamically predict the long-term CSS of DLBCL patients and compared the performance of the nomogram with traditional IPI and R-IPI prognostic scoring systems based on a large cohort of patients from the Surveillance, Epidemiology, and End Results (SEER) database.

## Materials and Methods

### Data Sources

Study data were retrieved from the SEER database, which collected patient-level clinical, pathological, demographic, therapeutic, and survival information from a total of 18 population-based cancer registries in the United States (http://seer.cancer.gov/). Patient consent and institutional review board approval were not required but a data use agreement was submitted to SEER database for access to data.

### Patient Selection

A case listing session was created from SEER database using SEER*Stat software (Version 8.3.6).

The following information on patient demographics, diagnosis, treatment, and survival were collected for analysis: patient identification, age at diagnosis, sex, race, year of diagnosis, marital status [married, single, or divorced/separated/widowed (DSW)], Ann Arbor stage, histology based on ICD-0–3, primary site, presence of systemic symptoms, IPI, cancer-directed surgery, radiation therapy, chemotherapy, vital status, cause of death, and survival months. In SEER database, Code the pretreatment point value for the IPI score as documented by the clinician in the range 000 (0 points) to 005 (5 points). Use a code in the 990 to 993 range if a risk category is described and points are not stated.

Study inclusion criteria were as follows: (1) Patients diagnosed with DLBCL-NOS according to the International Classification of Diseases for Oncology, third edition (ICD-O-3) histology code 9680 (DLBCL-NOS); (2) Patients diagnosed from 2004 to 2015, because we aimed to build a nomogram for predicting CSS in the rituximab era and 79% of DLBCL patients have received rituximab as first-line treatment from 2002 (16), however, access to the IPI relevant information (IPI score or risk stratification) was available from 2004 in the SEER database; (3) IPI relevant information was known; (4) diagnosed with DLBCL as the primary malignancy; (5) only one malignant tumor present.

The exclusion criteria were as follows: (1) those cases diagnosed without histological confirmation or diagnosed by autopsy or death certificate; (2) no active follow-up or survival time were recorded as 0 month; (3) Patients with missing data on any of these characteristics: primary site, IPI score, Ann Arbor Stage, B symptoms, surgery treatment, marital status and race; (4) patients with primary central nervous system or mediastinal DLBCL. Because they have unique clinical features, prognosis, and management.

**Figure 1:**
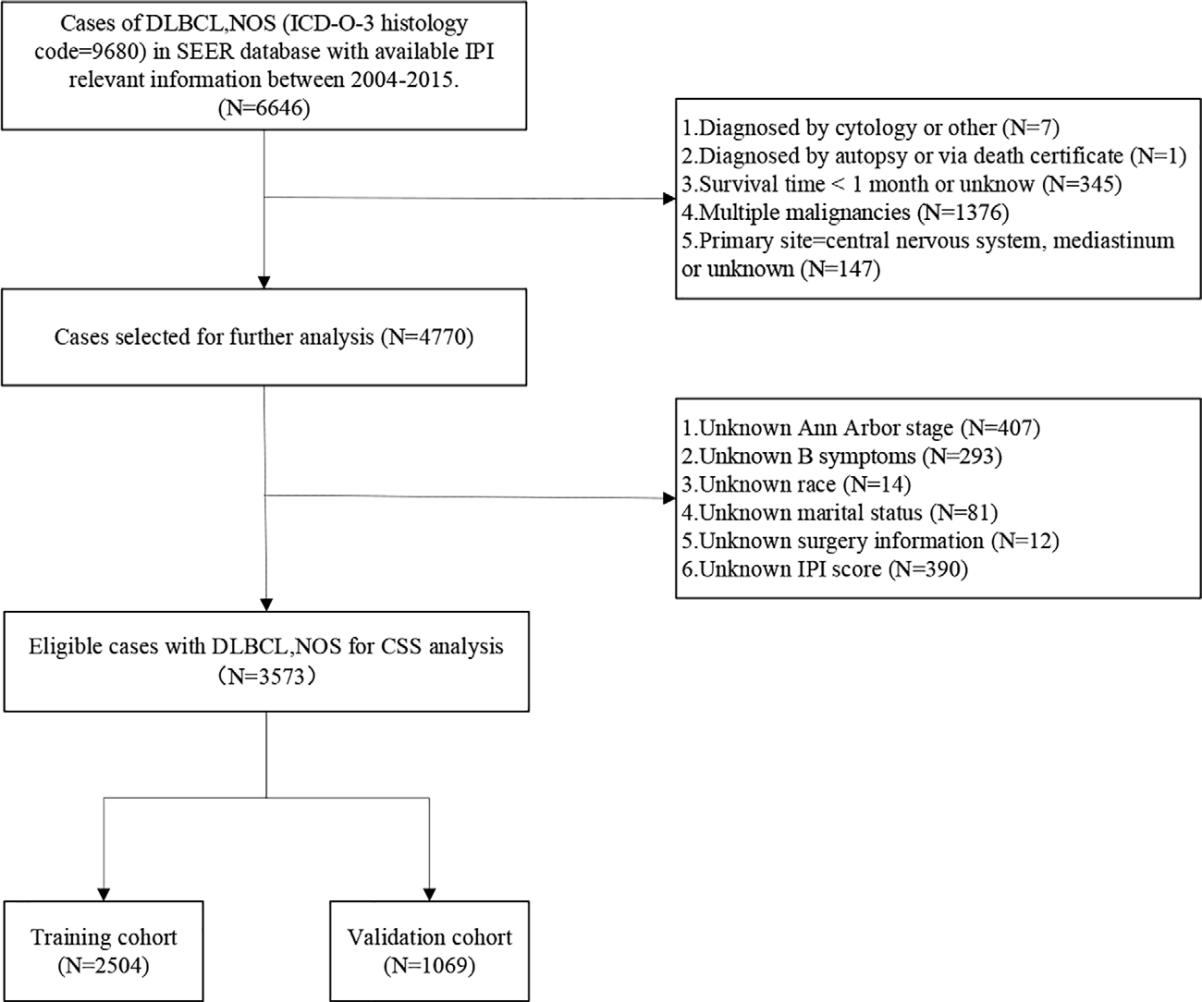
Flow diagram describing the evaluation and selection of patients with DLBCL.

A total of 130,896 patients diagnosed with DLBCL-NOS from 1975 to 2016 were extracted from the SEER database. However, only 6,646 patients with available IPI information from 2004 to 2015 were identified. After data screening, a total of 3,573 patients were eventually included in the present study. The process of patient selection was illustrated in **Figure 1**.

### Construction and Validation of the Nomogram

To improve the prediction power of the nomogram, 3,573 patients were randomly assigned to the training cohort (n = 2,504) and the validation cohort (n = 1,069) at a ratio of 7:3. The training cohort was used for construction and internal validation of the nomogram, and the validation cohort was applied for external validation.

In the first step, cox proportional hazard regression models were applied for univariable and multivariable analysis. Significant predictors of CSS determined by multivariate analyses were used to construct the nomogram. Then, the nomogram performance for predicting survival outcomes was evaluated with discrimination and calibration in internal validation and external validation. The discrimination ability was measured by calculating the C‐index, which resembles the area under ROC curve (AUC) value. A calibration plot was then used to show the concordance between predicted and observed probabilities for survival. Nomograms were subjected to 1,000 bootstrap resamples for internal validation in the training cohort and validation cohort, respectively.

### Nomogram Performance in Risk Stratification

Each patient was assessed a total score predicted by the nomogram. X-tile plots were constructed to assess nomogram score using four groups, and the most effective cutoff points were identified following correction for the use of minimum P statistics by Miller-Siegmund P-value correction (17). Receiver operating characteristic (ROC) curves and Kaplan-Meier survival curves were used to compare the precision of the survival prediction of the nomogram with IPI, R-IPI and Ann Arbor stage.

### Statistical Analysis

The endpoint of our study was CSS, which was defined as the time from diagnosis to death attributed to DLBCL. Overall survival (OS) was defined as the time from diagnosis to death from any cause. Patients who were alive at the time of last follow-up were counted as censored observations. Statistical analyses were performed using SPSS version 25.0 (IBM Corp., Armonk, NY, USA), GraphPad Prism version 8.0 (GraphPad, San Diego, CA, USA), and R version 3.6.1 (http://www.R-project.org). Demographic and clinical variables were compared between the training and validation cohorts using Pearson Chi-square test for categorical variables and independent t-test for continuous variables. Survival curves were estimated with the Kaplan-Meier method and compared with a log-rank test stratified according to the prognostic factors. Univariate and multivariate analyses were performed using the Cox regression model. Nomogram construction and validation were performed with Iasonos’ guide (18). Nomogram and calibration plots were constructed by R software using the “rms package.” The “shiny” and “DynNom” packages were used to develop the web-based survival rate calculator (https://www.shinyapps.io/), which can individually and dynamically predict the survival rates of patients. Statistical tests were two-sided, and P < 0.05 was considered statistically significant.

## Results

### Baseline Characteristics of Study Population

The baseline characteristics of the patients in the training and validation cohorts are presented in **Table 1**. The median age of all patients was 63 years (range, 9–97 years). Overall, there were no substantive differences between the two cohorts.

**Table 1:** Demographic and clinical characteristics of patients in the training and validation cohorts.

Variables		All patients, n (%)	Training cohort,n (%)	Validation cohort,n (%)	P-value
Entire cohort		3,573 (100.0)	2,504 (70.0)	1,069 (30.0)	
Age, years					0.554
≤39		390 (10.9)	281 (11.2)	109 (10.2)	
40–64		1,528 (42.8)	1,075 (42.9)	453 (42.4)	
≥65		1,655 (46.3)	1,148 (45.9)	507 (47.4)	
Sex					0.164
Female		1,533 (42.9)	1,055 (42.1)	478 (44.7)	
Male		2,040 (57.1)	1,449 (57.9)	591 (55.3)	
Race					0.906
White		3,006 (84.1)	2,111 (84.3)	895 (83.7)	
Black		256 (7.2)	177 (7.1)	79 (7.4)	
Other		311 (8.7)	216 (8.6)	95 (8.9)	
Marital status					0.309
Married		2,194 (61.4)	1,558 (62.2)	636 (59.5)	
Single		629 (17.6)	431 (17.2)	198 (18.5)	
DSW		750 (21.0)	515 (20.6)	235 (22.0)	
Primary site					0.521
Nodal		2,633 (73.7)	1,837 (73.4)	796 (74.5)	
Extranodal		940 (26.3)	667 (26.6)	273 (25.5)	
Ann Arbor stage					0.917
Stage I		669 (18.7)	465 (18.6)	204 (19.1)	
Stage II		797 (22.3)	565 (22.6)	232 (21.7)	
Stage III		773 (21.6)	537 (21.4)	236 (22.1)	
Stage IV		1,334 (37.4)	937 (37.4)	397 (37.1)	
B symptoms					0.24
No		2,213 (61.9)	1,567 (62.6)	646 (60.4)	
Yes		1,360 (38.1)	937 (37.4)	423 (39.6)	
Chemotherapy					0.787
No/unknown		252 (7.1)	179 (7.1)	73 (6.8)	
Yes	3,321 (92.9)		2,325 (92.9)	996 (93.2)	
Radiation					0.679
No		2,745 (76.8)	1,929 (77.0)	816 (76.3)	
Yes		828 (23.2)	575 (23.0)	253 (23.7)	
Primary Site Surgery					0.046
No		2,577 (72.1)	1,831 (73.1)	746 (69.8)	
Yes		996 (27.9)	673 (26.9)	323 (30.2)	
IPI					0.766
Low		1,248 (34.9)	869 (34.7)	379 (35.4)	
Low- intermediate		861 (24.1)	615 (24.5)	246 (23.0)	
High- intermediate		816 (22.8)	565 (22.6)	251 (23.5)	
High		648 (18.2)	455 (18.2)	193 (18.1)	
R-IPI					0.755
Very good		511 (14.3)	365 (14.6)	146 (13.7)	
Good		1,598 (44.7)	1,119 (44.7)	479 (44.8)	
Poor		1,464 (41.0)	1,020 (40.7)	444 (41.5)	

### Overall Survival and Cancer-Specific Survival of Patients

**Figure 2:**
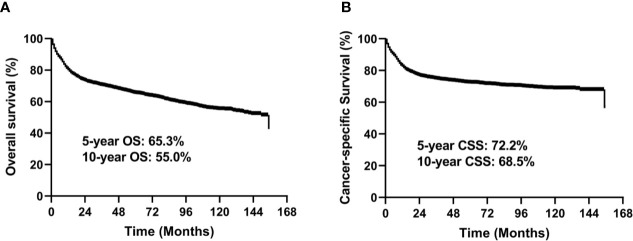
Kaplan-Meier survival curves for OS **(A)** and CSS **(B)** for all 3,573 patients.

In the entire study cohort, the median follow-up time was 49 months (range, 1–155 months). There were 1,342 deaths during the follow-up period, of which 979 were cancer-specific deaths and 363 deaths were from other causes. The 5-year OS rate was 65.3% and 5-year CSS rate was 72.2% for the entire group (**Figure 2**).

### Univariate and Multivariate Analyses of Cancer-Specific Survival in the Training Cohort

In the univariate analysis, the prognostic factors that affected survival in the training cohort were as follows: Age, sex, marital status, Ann Arbor stage, B symptom, chemotherapy, radiation therapy, and primary site surgery. Multivariate analysis revealed that Age, sex, marital status, Ann Arbor stage, B symptom, and chemotherapy were independent risk factors for CSS (**Table 2**).

**Table 2:** Univariable and multivariable analyses of cancer-specific survival in the training cohort.

Variables	Cancer-specific survival			Multivariate analysis			
	Univariate analysis						
	HR	95% CI	P-value	HR	95% CI	P-value	Nomogramscore
Age, years							
≤39	Ref			Ref			0
40–64	2.648	1.766–3.971	**P < 0.001**	2.623	1.737–3.963	**0.001**	60
≥65	5.088	3.422–7.565	**P < 0.001**	4.983	3.297–7.533	**P < 0.001**	100
Sex							
Female	Ref			Ref			0
Male	1.209	1.035–1.412	**0.016**	1.377	1.172–1.617	**P < 0.001**	20
Race							
White	Ref			Ref			
Black	1.113	0.838–1.479	0.459				
Other	0.984	0.744–1.301	0.909				
Marital status							
Married	Ref			Ref			0
Single	1.057	0.859–1.301	0.601	1.452	1.17–1.803	**0.001**	24
DSW	1.331	1.109–1.597	**0.002**	1.176	0.972–1.422	0.096	10
Ann Arbor stage							
Stage I	Ref			Ref			0
Stage II	1.673	1.218–2.298	**0.001**	1.692	1.224–2.338	**0.001**	35
Stage III	2.578	1.904–3.489	**P < 0.001**	2.377	1.727–3.272	**P < 0.001**	57
Stage IV	3.757	2.846–4.961	**P < 0.001**	3.442	2.563–4.621	**P < 0.001**	80
B symptoms							
No	Ref			Ref			0
Yes	1.460	1.254–1.699	**P < 0.001**	1.330	1.135–1.559	**P < 0.001**	18
Chemotherapy							
No/unknown	Ref			Ref			72
Yes	0.366	0.292–0.457	**P < 0.001**	0.313	0.247–0.396	**P < 0.001**	0
Radiation							
No	Ref			Ref			
Yes	0.597	0.488–0.732	**P < 0.001**	0.900	0.728–1.113	0.330	
Primary Site Surgery							
No	Ref			Ref			
Yes	0.772	0.646–0.923	**0.004**	0.849	0.708–1.017	0.076	

Bold values indicate P<0.05 in univariate analysis and multivariate analysis.

### Construction and Validation of the Nomogram

**Figure 3:**
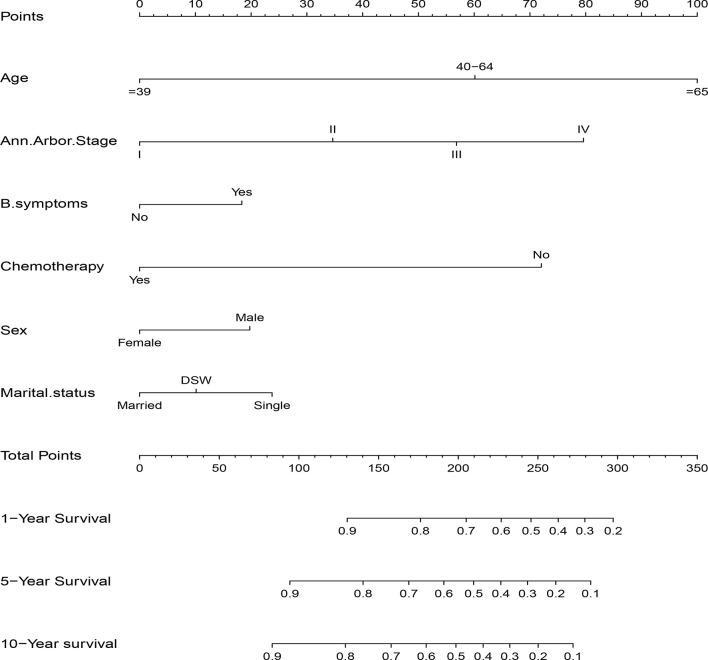
Nomogram predicting 1-, 5-, and 10-year CSS of patients with DLBCL.

The significant independent prognostic factors of CSS for DLBCL were used to construct the nomogram. The nomogram exhibited that age made the largest contribution to survival outcome, followed by Ann Arbor stage, chemotherapy, marital status, sex, and B symptom (**Figure 3**).

**Figure 4:**
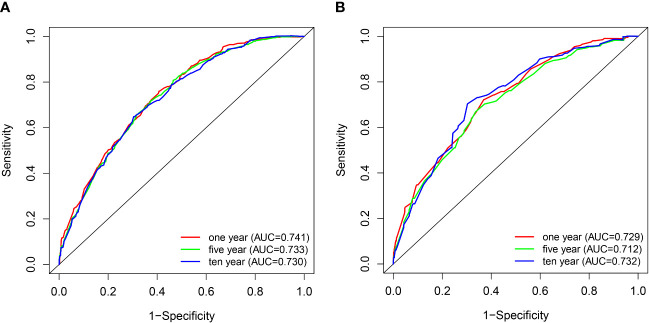
ROC curves of the nomogram for 1-, 5-, and 10-year CSS prediction in the training cohort **(A)** and validation cohort **(B)**.

**Figure 5:**
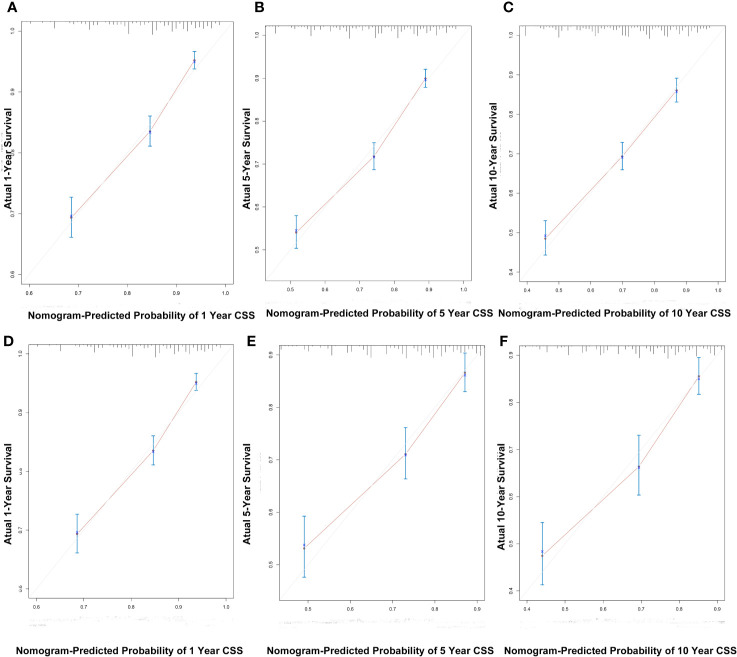
The calibration curves for predicting patient CSS at 1-, 5-, and 10-years in the training cohort **(A–C)** and validation cohort **(D–F)**.

C index of the nomogram was calculated to be 0.709 (95% CI, 0.692–0.726) in the training cohort and 0.700 (95% CI, 0.671–0.729) in the validation cohort. Similarly, AUC values of the nomogram for predicting the 1-, 5-, and 10- year CSS rates in the training cohort were 0.741, 0.733, and 0.730, respectively (**Figure 4A**). These values were 0.729, 0.712, and 0.732 in the validation cohort (**Figure 4B**). In addition, the calibration curves for the probability of the 1-, 5- and 10-year CSS showed favorable agreement between the actual observed outcome and the prediction by the nomogram both in the training (**Figures 5A–C**) and validation cohorts (**Figures 5D–F**).

These findings indicated that the discriminatory capacity and accuracy of the nomogram was reasonably clear.

### Dynamic Web-Based Survival Rate Calculator

**Figure 6:**
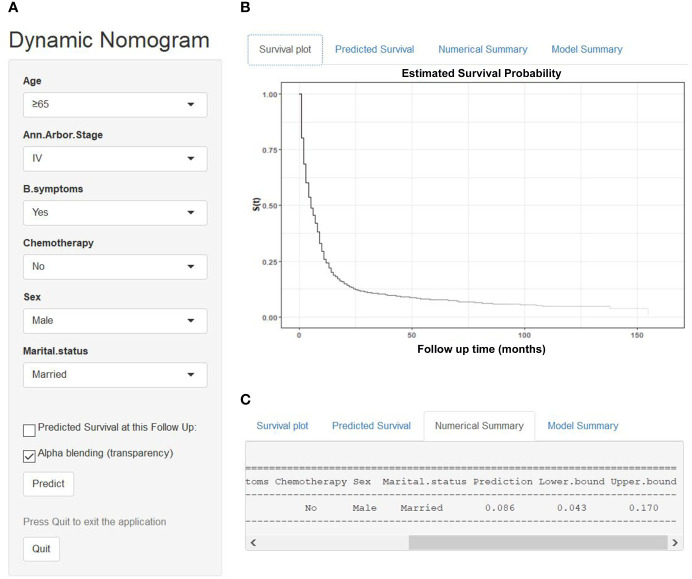
An example to illustrate the usage of the web-based survival rate calculator. **(A)** A 66-year-old married man was diagnosed as DLBCL with Ann Arbor stage of IV, presented with B symptoms, refused chemotherapy, **(C)** his 5-year cancer-specific survival rate is approximately 8.6% (95% CI 4.3–17.0%). **(B)** His survival plot according to the web survival rate calculator.

We established a dynamic web-based survival rate calculator (https://qiaofengzhong.shinyapps.io/DynNomapp/) to predict the long-term CSS of patients with DLBCL based on the nomogram. For example, a 66-year-old married man was diagnosed as DLBCL with Ann Arbor stage of IV, presented with B symptoms, if he refused chemotherapy, his 5-year cancer-specific survival rate is approximately 8.6% (95% CI 4.3–17.0%) (**Figure 6**).

### Nomogram Performance in Risk Stratification

The training and validation cohorts were each stratified into four groups based on the total points predicted by the nomogram for further evaluate the calibration. We defined optimal cutoffs value based on the X-tile plots. According to the cut‐off values, patients were classified into low risk (n = 898, 25.1%; score <119), low-intermediate risk (n = 949, 26.6%; score 119–157), high-intermediate risk (n = 844, 23.6%; score 158–187), and high risk (n = 882, 24.7%; score >187).

### Comparison of the Predictive Accuracy Between the Novel Risk Stratification Model and Conventional Prognostic Scoring Systems

We compared the predictive accuracy for CSS between the nomogram and the traditional prognostic scoring systems by the values of AUC and the K-M survival curves.

**Figure 7:**
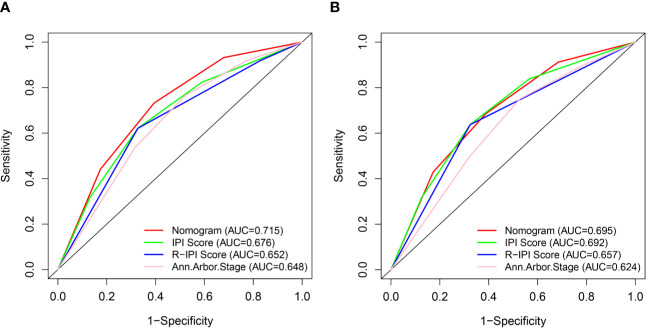
ROC curves of the nomogram and IPI, R-IPI, and Ann Arbor stage prognostic systems for 5-year CSS prediction in the training **(A)** and validation cohort **(B)**.

With regard to the prediction of 5-year CSS rates for DLBCL, higher AUC values were observed in the nomogram risk stratification model (0.715) compared with the IPI (0.676), R-IPI (0.652), and Ann Arbor stage (0.648) (**Figure 7A**). Similarly, in the validation cohort, the AUC values of the IPI (0.692), R-IPI (0.657), and Ann Arbor stage (0.624) were lower than that of the nomogram risk stratification model (0.695) (**Figure 7B**).

**Figure 8:**
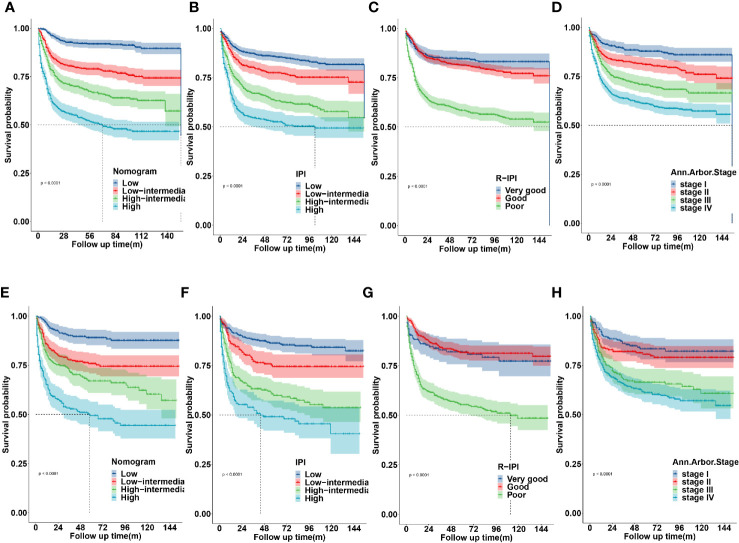
Kaplan-Meier survival curves according to the nomogram risk level, IPI, R-IPI, and Ann arbor stage in the training **(A–D)** and validation cohort **(E–H)**.

The novel prognostic model displayed better accuracy than IPI, R-IPI, and Ann Arbor stage in both cohorts (**Figures 8A, E**). IPI was unsatisfactory for the stratification of patients with low and low-intermediate, high and high- intermediate in both sets (**Figures 8B, F**). R-IPI was unsatisfactory for discriminating between good and very good patient groups in both cohorts (**Figures 8C, G**). Ann Arbor stage was unsatisfactory for the stratification of patients with stage I and II, stage III and IV disease in both sets (**Figures 8D, H**).

These results revealed that the established nomogram risk stratification model indicated greater discriminatory capacity and accuracy in CSS prediction of DLBCL compared with the IPI, R-IPI, and Ann Arbor stage.

## Discussion

DLBCL is one of the most common subtypes of NHL with great heterogeneity in clinical behavior and response to treatment. Precision medicine rapidly developed in recent years. Clinicians must establish individualized treatment and follow-up strategies for patients, which requires more accurate and individual survival prognostic models. Although the IPI and its permutations (R-IPI and NCCN-IPI) are still recognized as good prognostic models for DLBCL, they mainly reflect a stationary state before treatment and cannot consistently predict prognosis for individual patients. A variety of new biomarkers and gene signatures with prognostic significance for risk stratification for DLBCL also has been identified, such as cell of origin (19), C-MYC, BCL-2, or BCL-6 rearrangements (20), MYC/BCL2 expression (21), PD-1 and PD-L1 expression (22, 23), absolute lymphocyte/monocyte count (24–26), which have all shown promise as new predictive factors. However, most of these parameters remain expensive and not readily accessible. Nomograms have become more widely used prognostic tools in recent years as it permits improved predictive accuracy for clinical outcomes compared with the former prognostic indexes through calculating the cumulative effect of each independent variable (18). Nomograms were previously developed for DLBCL (27–35), although some of these nomograms incorporated clinicopathological parameters into nomogram construction for accurate survival prediction, the generalizable conclusion of these study were drawn based on a relatively small patient cohort, and most of them have not yet been validated in an external cohort. In addition, these nomograms may be not easily applied in daily clinical practice due to the need to perform manual calculations. Therefore, we developed a dynamic web-based survival rate calculator (https://qiaofengzhong.shinyapps.io/DynNomapp/) to predict the long-term survival of patients with DLBCL based on the nomogram. It can dynamically predict the CSS rate of patients at different time points. In the present study, a nomogram was developed based on 3,573 cases from the SEER database. We identified six demographic, clinicopathological and therapeutic features including age at diagnosis, sex, marital status, Ann Arbor stage, B symptom, and chemotherapy as independent predictors of CSS in patients with DLBCL, which were used to construct the nomogram. These simple parameters are routinely recorded, easily available in daily clinical practice, and are inexpensive to test. The nomogram exhibited that age made the largest contribution to survival outcome for DLBCL. Age has been demonstrated to be a significant predictive and prognostic factor in previous studies (36, 37). The NCCN-IPI additionally emphasized the role of age by allocating three points to very elderly patients. The correlation of age with increased mortality was not surprising as patients of advanced age tend to have comorbidities, poorer ECOG and therapy-related toxicity compared to younger patients, which might contribute to less-effective outcome.

Ann Arbor stage also had a strong prognostic association with CSS for DLBCL. This observation was similar to previous reports and was not unexpected as advanced stage indicates disseminated disease.

Therapeutic factors play an important role in individual survival of cancer patients. It is worth noting that with the inclusion of chemotherapy, which usually convey a tremendous survival benefit in DLBCL, the novel nomogram is only marginally better than conventional R-IPI. One possible reason for this was that the real impact of chemotherapy was underestimated. In the SEER database, the chemotherapy field is coded to “Yes” or “No/Unknown,” however, the detailed regimens and cycles were unavailable. Therefore, the findings of our study should be interpreted cautiously.

Interestingly, this study showed that married patients were more likely to have a good survival outcome than those who were unmarried or DSW. Numerous published studies addressed the association between marital status and disease specific survival, with variable results. A systematic review that included a total of 18 studies confirmed that unmarried, DSW patients diagnosed with any of the malignancies are more likely to present with advanced stage disease (38). Although the real mechanisms underlying the association between marital status and cancer specific survival are still largely unknown. There are several possible reasons that may help to explain this. Firstly, married patients may have better health insurance and socioeconomic status compared to single and DSW patients. Secondly, widowed patients tend to be older, which may increase cancer mortality. Thirdly, married patients tend to have better adherence with prescribed treatments than unmarried patients (39). Impaired adherence has been associated with poorer outcomes in patients with cancer. Lastly, patients who are married display less distress, depression, and anxiety than their unmarried counterparts after a diagnosis of cancer, as a partner can share the emotional burden and provide appropriate social support (40).

It is worth noting that, the results of **Table 2** showed that “single” marital status was not prognostic in the univariate analysis, however, it was of significance in the multivariate analysis, the possible reason was that there may be a certain correlation between marital status and other confounding factors, after the influence of other factors is eliminated by multivariate analysis, it is found that “single” marital status plays an independent role in cancer prognosis.

In addition, the nomogram exhibited that the female had a better survival outcome than male. Multiple randomized trials (RICOVER 60, NHL-B2, MInT, and the MegaCHOEP trials) also have demonstrated superior outcomes in female relative to male, particularly in older women. Two factors may account for this result: (1) elderly females had a statistically significant slower clearance of rituximab, which brought about longer exposure time and higher serum levels of rituximab in the body (41, 42). (2) Women had stronger humoral and cellular immunity than men (43).

The B symptoms has prognostic significance. B symptoms tend to correlate with disease that is either more widespread or of a higher histologic grade (44). In line with this, B symptoms was an independent risk factor for poor prognosis in this study.

A risk stratification model was generated on the basis of patient’s total scores evaluated by the nomogram, which had a favorable level of predictive accuracy compared to the IPI, R-IPI, and Ann Arbor stage. Although the traditional prognostic scoring systems have retained prognostic significance regarding CSS for DLBCL patients in our results, a limitation existed that they simply suggest the general prognostic value of a patient without dynamic survival estimate and risk stratification. In addition, IPI reflects pretreatment factors relating to prognosis, whereas our new prognostic model included covariates both from the baseline and after treatment, thus adding the strength of treatment-responsiveness in prognostication. Moreover, the new prognostic score could divide DLBCL patients into four groups more accurately, which could help decrease over-therapy in low-risk patients and promote effective therapies in high risk patients to prevent the fatal nature of this disease. Therefore, we have developed a new prediction model in the rituximab era that may provide an accurate risk stratification of individual DLBCL patient to determine the optimal treatment and follow-up strategies. To our knowledge, this is the first large-cohort, comprehensive retrospective study that has developed a nomogram and a web-based survival rate calculator to predict the CSS in DLBCL and defined optimal cutoff values based on the nomogram score for risk stratification compared to classic prognosis indexes from the SEER database in the rituximab era.

There are several unavoidable limitations in the present research. Firstly, the SEER database was lacking of detail information of ECOG PS, LDH, NCCN-IPI, molecular markers, gene expression, chemotherapy regimens, and complications that may affect prognosis. We are unable to further analyze the impact of these potential prognostic factors and compare this nomogram model with NCCN-IPI. Secondly, due to the retrospective nature of the study and the strict inclusion and exclusion criteria used, potential selection biases might be present. Thirdly, although our nomogram model was developed and validated in two independent subgroups of patients from the SEER database, external validation of the predictive model is still necessary. Lastly, although this nomogram model predicted well in patients with lower-risk, its predictive ability was reduced in high-risk patients. Therefore, the findings of this study need to be confirmed by a larger and prospective cohort of DLBCL patients.

In conclusion, we have developed a predictive nomogram and a web-based survival rate calculator by using some clinically common variables from the SEER database that can predict individual and dynamic CSS for DLBCL patients with a high degree of accuracy based on a large number of patients in the rituximab era. The proposed novel nomogram risk stratification model showed a better level of risk discrimination and predictive accuracy than IPI, R-IPI, and Ann Arbor stage, which may help guide individual treatment and follow-up strategies.

## Data Availability Statement

The raw data supporting the conclusions of this article will be made available by the authors, without undue reservation.

## Ethics Statement

Patient consent and institutional review board approval were not required, but a data use agreement was submitted to SEER database for access to data.

## Author Contributions

YS conceived the concept, designed the research, drafted and reviewed the article. QZ extracted and analyzed the data and wrote the manuscript. All authors contributed to the article and approved the submitted version.

## Funding

This work was supported by grants of the Beijing Natural Science Foundation, Grant/Award Number: H2018206591.

## Conflict of Interest

The authors declare that the research was conducted in the absence of any commercial or financial relationships that could be construed as a potential conflict of interest.

## Abbreviations

CSS, cancer-specific survival; DLBCL, diffuse large B-cell lymphoma; SEER, Surveillance, Epidemiology and End Results; NHL, non-Hodgkin lymphoma; IPI, International Prognostic Index; R‐IPI, Revised‐IPI; NCCN-IPI, National Comprehensive Cancer Network IPI; ECOGPS, Eastern Cooperative Oncology Group performance status; LDH, lactate dehydrogenase; DLBCL-NOS, diffuse large B-cell lymphoma, not otherwise specified; ICD-O-3, International Classification of Diseases for Oncology, third edition; DSW, divorced/separated/widowed; ROC, Receiver operating characteristic; AUC, area under ROC curve; OS, Overall survival, HR, Hazard ratio; CI, confidence interval.

## References

[1] Lenz G Staudt LM Aggressive lymphomas. N Engl J Med (2010) 362:1417–29. 10.1056/NEJMra0807082 PMC7316377 20393178

[2] Nogai H Dorken B Lenz G Pathogenesis of non-Hodgkin’s lymphoma. J Clin Oncol (2011) 29:1803–11. 10.1200/JCO.2010.33.3252 21483013

[3] Coiffier B Lepage E Briere J Herbrecht R Tilly H Bouabdallah R CHOP chemotherapy plus rituximab compared with CHOP alone in elderly patients with diffuse large-B-cell lymphoma. N Engl J Med (2002) 346:235–42. 10.1056/NEJMoa011795 11807147

[4] Friedberg JW Relapsed/refractory diffuse large B-cell lymphoma. Hematol Am Soc Hematol Educ Program (2011) 2011:498–505. 10.1182/asheducation-2011.1.498 22160081

[5] International Non-Hodgkin’s Lymphoma Prognostic Factors P A predictive model for aggressive non-Hodgkin’s lymphoma. N Engl J Med (1993) 329:987–94. 10.1056/NEJM199309303291402 8141877

[6] Sehn LH Berry B Chhanabhai M Fitzgerald C Gill K Hoskins P The revised International Prognostic Index (R-IPI) is a better predictor of outcome than the standard IPI for patients with diffuse large B-cell lymphoma treated with R-CHOP. Blood (2007) 109:1857–61. 10.1182/blood-2006-08-038257 17105812

[7] Zhou Z Sehn LH Rademaker AW Gordon LI Lacasce AS Crosby-Thompson A An enhanced International Prognostic Index (NCCN-IPI) for patients with diffuse large B-cell lymphoma treated in the rituximab era. Blood (2014) 123:837–42. 10.1182/blood-2013-09-524108 PMC5527396 24264230

[8] Wu G Keating A Biomarkers of potential prognostic significance in diffuse large B-cell lymphoma. Cancer (2006) 106:247–57. 10.1002/cncr.21586 16342164

[9] Hong F Kahl BS Gray R Incremental value in outcome prediction with gene expression-based signatures in diffuse large B-cell lymphoma. Blood (2013) 121:156–8. 10.1182/blood-2012-08-450106 23160463

[10] Vaidya R Witzig TE Prognostic factors for diffuse large B-cell lymphoma in the R(X)CHOP era. Ann Oncol (2014) 25:2124–33. 10.1093/annonc/mdu109 PMC4288137 24625454

[11] Wight JC Chong G Grigg AP Hawkes EA Prognostication of diffuse large B-cell lymphoma in the molecular era: moving beyond the IPI. Blood Rev (2018) 32:400–15. 10.1016/j.blre.2018.03.005 29605154

[12] Schmitz R Wright GW Huang DW Johnson CA Phelan JD Wang JQ Genetics and Pathogenesis of Diffuse Large B-Cell Lymphoma. N Engl J Med (2018) 378:1396–407. 10.1056/NEJMoa1801445 PMC6010183 29641966

[13] Gao R Liang JH Wang L Zhu HY Wu W Cao L Low serum cholesterol levels predict inferior prognosis and improve NCCN-IPI scoring in diffuse large B cell lymphoma. Int J Cancer (2018) 143:1884–95. 10.1002/ijc.31590 29744861

[14] Chapuy B Stewart C Dunford AJ Kim J Kamburov A Redd RA Molecular subtypes of diffuse large B cell lymphoma are associated with distinct pathogenic mechanisms and outcomes. Nat Med (2018) 24:679–90. 10.1038/s41591-018-0016-8 PMC6613387 29713087

[15] Balachandran VP Gonen M Smith JJ DeMatteo RP Nomograms in oncology: more than meets the eye. Lancet Oncol (2015) 16:e173–80. 10.1016/S1470-2045(14)71116-7 PMC4465353 25846097

[16] Link BK Brooks J Wright K Pan X Voelker M Chrischilles E Diffuse large B-cell lymphoma in the elderly: diffusion of treatment with rituximab and survival advances with and without anthracyclines. Leuk Lymphoma (2011) 52:994–1002. 10.3109/10428194.2011.557167 21338277 PMC3601377

[17] Camp Robert L Dolled-Filhart M Rimm David L X-tile: a new bio-informatics tool for biomarker assessment and outcome-based cut-point optimization. Clin Cancer Res (2004) 10:7252–9. 10.1158/1078-0432.CCR-04-0713 15534099

[18] Iasonos A Schrag D Raj GV Panageas KS How to build and interpret a nomogram for cancer prognosis. J Clin Oncol (2008) 26:1364–70. 10.1200/JCO.2007.12.9791 18323559

[19] Rosenwald A Wright G Chan WC Connors JM Campo E Fisher RI The use of molecular profiling to predict survival after chemotherapy for diffuse large-B-cell lymphoma. N Engl J Med (2002) 346:1937–47. 10.1056/NEJMoa012914 12075054

[20] Rosenthal A Younes A High grade B-cell lymphoma with rearrangements of MYC and BCL2 and/or BCL6: Double hit and triple hit lymphomas and double expressing lymphoma. Blood Rev (2017) 31:37–42. 10.1016/j.blre.2016.09.004 PMC5572137 27717585

[21] Hu S Xu-Monette ZY Tzankov A Green T Wu L Balasubramanyam A MYC/BCL2 protein coexpression contributes to the inferior survival of activated B-cell subtype of diffuse large B-cell lymphoma and demonstrates high-risk gene expression signatures: a report from The International DLBCL Rituximab-CHOP Consortium Program. Blood (2013) 121:4021–31; quiz 250. 10.1182/blood-2012-10-460063 PMC3709650 23449635

[22] Kiyasu J Miyoshi H Hirata A Arakawa F Ichikawa A Niino D Expression of programmed cell death ligand 1 is associated with poor overall survival in patients with diffuse large B-cell lymphoma. Blood (2015) 126:2193–201. 10.1182/blood-2015-02-629600 PMC4635115 26239088

[23] Kwiecinska A Tsesmetzis N Ghaderi M Kis L Saft L Rassidakis GZ CD274 (PD-L1)/PDCD1 (PD-1) expression in de novo and transformed diffuse large B-cell lymphoma. Br J Haematol (2018) 180:744–8. 10.1111/bjh.14432 27879989

[24] Kim DH Baek JH Chae YS Kim YK Kim HJ Park YH Absolute lymphocyte counts predicts response to chemotherapy and survival in diffuse large B-cell lymphoma. Leukemia (2007) 21:2227–30. 10.1038/sj.leu.2404780 17554383

[25] Lin B Chen C Qian Y Feng J Prognostic role of peripheral blood lymphocyte/monocyte ratio at diagnosis in diffuse large B-cell lymphoma: a meta-analysis. Leuk Lymphoma (2015) 56:2563–8. 10.3109/10428194.2015.1014367 25686648

[26] Tadmor T Bari A Sacchi S Marcheselli L Liardo EV Avivi I Monocyte count at diagnosis is a prognostic parameter in diffuse large B-cell lymphoma: results from a large multicenter study involving 1191 patients in the pre- and post-rituximab era. Haematologica (2014) 99:125–30. 10.3324/haematol.2013.088161 PMC4007925 23935023

[27] Han Y Yang J Liu P He X Zhang C Zhou S Prognostic Nomogram for Overall Survival in Patients with Diffuse Large B-Cell Lymphoma. Oncologist (2019) 24:e1251–e61. 10.1634/theoncologist.2018-0361 PMC6853087 30952824

[28] Zhong H Chen J Cheng S Chen S Shen R Shi Q Prognostic nomogram incorporating inflammatory cytokines for overall survival in patients with aggressive non-Hodgkin’s lymphoma. EBioMedicine (2019) 41:167–74. 10.1016/j.ebiom.2019.02.048 PMC6443577 30827933

[29] Go SI Park S Kang MH Kim HG Kim HR Lee GW Clinical impact of prognostic nutritional index in diffuse large B cell lymphoma. Ann Hematol (2019) 98:401–11. 10.1007/s00277-018-3540-1 30413902

[30] Wang Y Li J Wei R Liu C Nataraj A Yan J Prognostic Factors Associated With Bone Lymphoma Primarily Presenting in the Spine. Spine (Phila Pa 1976) (2019) 44:185–94. 10.1097/BRS.0000000000002844 30096126

[31] Go SI Park S Kim JH Kim HR Kim M Moon K A new prognostic model using the NCCN-IPI and neutrophil-to-lymphocyte ratio in diffuse large B-cell lymphoma. Tumori (2018) 104:292–9. 10.5301/tj.5000694 29737944

[32] Jiang S Qin Y Liu P Yang J Yang S He X A prognostic nomogram constructed for relapsed or refractory diffuse large B-cell lymphoma patients. Asia Pac J Clin Oncol (2019) Epub ahead of print. 10.1111/ajco.13222 31264371

[33] Jiang S Qin Y Liu P Yang J Yang S He X Prognostic Nomogram and Predictive Factors in Refractory or Relapsed Diffuse Large B-Cell Lymphoma Patients Failing Front-Line R-CHOP Regimens. J Cancer (2020) 11:1516–24. 10.7150/jca.36997 PMC6995391 32047558

[34] Lin JL Lin JX Li P Xie JW Wang JB Lu J Dynamic prediction of long-term survival in patients with primary gastric diffuse large B-cell lymphoma: a SEER population-based study. BMC Cancer (2019) 19:873. 10.1186/s12885-019-5993-6 31481021 PMC6724291

[35] Yin X Xu A Fan F Huang Z Cheng Q Zhang L Incidence and Mortality Trends and Risk Prediction Nomogram for Extranodal Diffuse Large B-Cell Lymphoma: An Analysis of the Surveillance, Epidemiology, and End Results Database. Front Oncol (2019) 9:1198. 10.3389/fonc.2019.01198 31781500 PMC6861389

[36] Zander T Wiedenmann S Wolf J Prognostic factors in Hodgkin’s lymphoma. Ann Oncol (2002) 13 Suppl 1:67–74. 10.1093/annonc/13.s1.67 12078906

[37] Moccia AA Donaldson J Chhanabhai M Hoskins PJ Klasa RJ Savage KJ International Prognostic Score in advanced-stage Hodgkin’s lymphoma: altered utility in the modern era. J Clin Oncol (2012) 30:3383–8. 10.1200/JCO.2011.41.0910 22869887

[38] Buja A Lago L Lago S Vinelli A Zanardo C Baldo V Marital status and stage of cancer at diagnosis: A systematic review. Eur J Cancer Care (Engl) (2018) 27undefined. 10.1111/ecc.12755 28850741

[39] Cohen SD Sharma T Acquaviva K Social support and chronic kidney disease: an update. Adv Chronic Kidney Dis (2007) 14:335–44. 10.1053/j.ackd.2007.04.007 17904500

[40] Goldzweig G Andritsch E Hubert A Brenner B Walach N Perry S Psychological distress among male patients and male spouses: what do oncologists need to know? Ann Oncol (2010) 21:877–83. 10.1093/annonc/mdp398 19822532

[41] Pfreundschuh M Muller C Zeynalova S Kuhnt E Wiesen MH Held G Suboptimal dosing of rituximab in male and female patients with DLBCL. Blood (2014) 123:640–6. 10.1182/blood-2013-07-517037. 10.1111/ecc.12755. 24297867

[42] Muller C Murawski N Wiesen MH Held G Poeschel V Zeynalova S The role of sex and weight on rituximab clearance and serum elimination half-life in elderly patients with DLBCL. Blood (2012) 119:3276–84. 10.1182/blood-2011-09-380949 22337718

[43] Bouman A Heineman MJ Faas MM Sex hormones and the immune response in humans. Hum Reprod Update (2005) 11:411–23. 10.1093/humupd/dmi008 15817524

[44] Anderson T Chabner BA Young RC Berard CW Garvin AJ Simon RM Malignant lymphoma. 1. The histology and staging of 473 patients at the National Cancer Institute. Cancer (1982) 50:2699–707. 10.1002/1097-0142(19821215)50:12<2699::aid-cncr2820501202>3.0.co;2-a 7139563

